# Prolonged neurologic deficits with brain MRI changes following ECT in an adolescent with a *CACNA1a*-related disorder; a case report

**DOI:** 10.1186/s12883-022-02994-7

**Published:** 2022-12-09

**Authors:** Joseph Vithayathil, Colbey Freeman, Marin Jacobwitz, Erin Simon Schwartz, Sonika Agarwal

**Affiliations:** 1 Division of Neurology, Children’s Hospital of Philadelphia, 3401 Civic Center Drive, Philadelphia, PA 19104 USA; 2 Department of Radiology, Hospital of the University of Pennsylvania, Philadelphia, USA; 3 Division of Neuroradiology, Children’s Hospital of Philadelphia, Philadelphia, USA

**Keywords:** Electroconvulsive therapy (ECT), CACNA1a, Status epilepticus, Cerebral edema, Hemiplegic migraine

## Abstract

**Background:**

Electroconvulsive therapy is used to treat depression and schizophrenia with infrequent use in pediatric patients. We report a case of an adolescent with autism spectrum disorder and acute catatonia that presented with status epilepticus (SE) and prolonged neurologic deficits with unilateral left cerebral edema on imaging following unilateral electroconvulsive therapy (ECT) on the right side, subsequently found to have a *CACNA1a* pathogenic variant. This case highlights a potential adverse effect of ECT in patients with CACNA1a related disorders.

**Case:**

The patient received unilateral ECT to the right side and subsequently had an episode of SE with right-sided hemiplegia for 72 h prior to regaining some function with persistent mild right-hand weakness that persisted for at least 1–2 weeks. A brain MRI 2 days after ECT was unremarkable, but a repeat MRI on day four of admission showed left hemisphere cortical diffusion restriction, increased perfusion and T2 prolongation suggestive of cortical edema. They had whole exome genetic testing sent after discharge that showed a known pathogenic *CACNA1a* variant (p.I1709T). *CACNA1a* encodes the P/Q type calcium channels and deleterious variants in this gene result in a channelopathy associated with a spectrum of neurodevelopmental disorders that include autism spectrum disorder, hemiplegic migraine with unilateral cerebral edema, epileptic encephalopathies, or episodic ataxia syndromes.

**Conclusion:**

A literature review of ECT and neurologic deficits showed that most neurologic deficits resolve within 30 min of ECT. Case reports of prolonged deficits are rare and there are no prior reports of acute MRI changes related to ECT. Thus, the acute deterioration and MRI findings in this patient are likely related to the underlying *CACNA1a* channelopathy disorder with ECT as a precipitating event. This case report suggests care should be taken when using ECT in patients with pathogenic variants in *CACNA1a*. Furthermore, it reinforces the utility and importance of expanded genetic testing in patients with neurodevelopmental disorders as findings can provide valuable information that can guide treatment decisions.

## Background

Electroconvulsive therapy (ECT) has been used since the 1930s to treat depression and schizophrenia [1]. In the US, around 100,000 patients receive ECT per year with less than 1% of these occurring in patients under 18 years of age [2, 3].

Given the relatively infrequent use of ECT in pediatric patients, it is important that adverse events related to this procedure are highlighted, particularly if specific disorders may incur a higher risk for complications related to ECT.

In this case report, we highlight the adverse effects of ECT in a patient with autism spectrum disorder that was subsequently found to have a de novo pathogenic *CACNA1a* variant that is known to be associated with hemiplegic migraines. Ultimately, the most likely diagnosis is that the patient had a severe hemiplegic migraine triggered by ECT. This case is important because it reports on an adverse event related to ECT and highlights the importance of expanded genetic testing in patients with autism spectrum disorders to help identify potential risks related to interventions such as ECT.

## Case

The patient is an adolescent with autism spectrum disorder who, at baseline, is high functioning with no apparent focal neurologic deficits and normal mental status. Three months prior to presentation, they had a subacute onset of neuropsychiatric symptoms consisting of disordered thinking, inappropriate statements, auditory hallucinations, and psychomotor retardation with progression to acute catatonia. They were admitted to an outside hospital after having symptoms for 6 weeks and had an extensive workup to evaluate for an organic etiology, including autoimmune, metabolic, and infectious causes. A lumbar puncture showed no evidence of infection or inflammation. Autoimmune encephalitis serum and cerebrospinal fluid (CSF) samples were negative. An MRI of their brain without contrast revealed only cerebellar atrophy, and an EEG was unremarkable. Following the negative workup, they were diagnosed with catatonia and primary psychiatric mood disorder. They had initial improvement with lorazepam and subsequently discharged, but this improvement was not sustained. They were followed by psychiatry and behavioral health, who recommended ECT as they were not tolerating increased doses of lorazepam. Other than autism spectrum disorder, the patient had no other prior medical issues or prior genetic testing. Family history is notable for a sibling with autism spectrum disorder. The patient lives with his parents and there were no known toxin exposures or notable travel history.

**Fig. 1:**
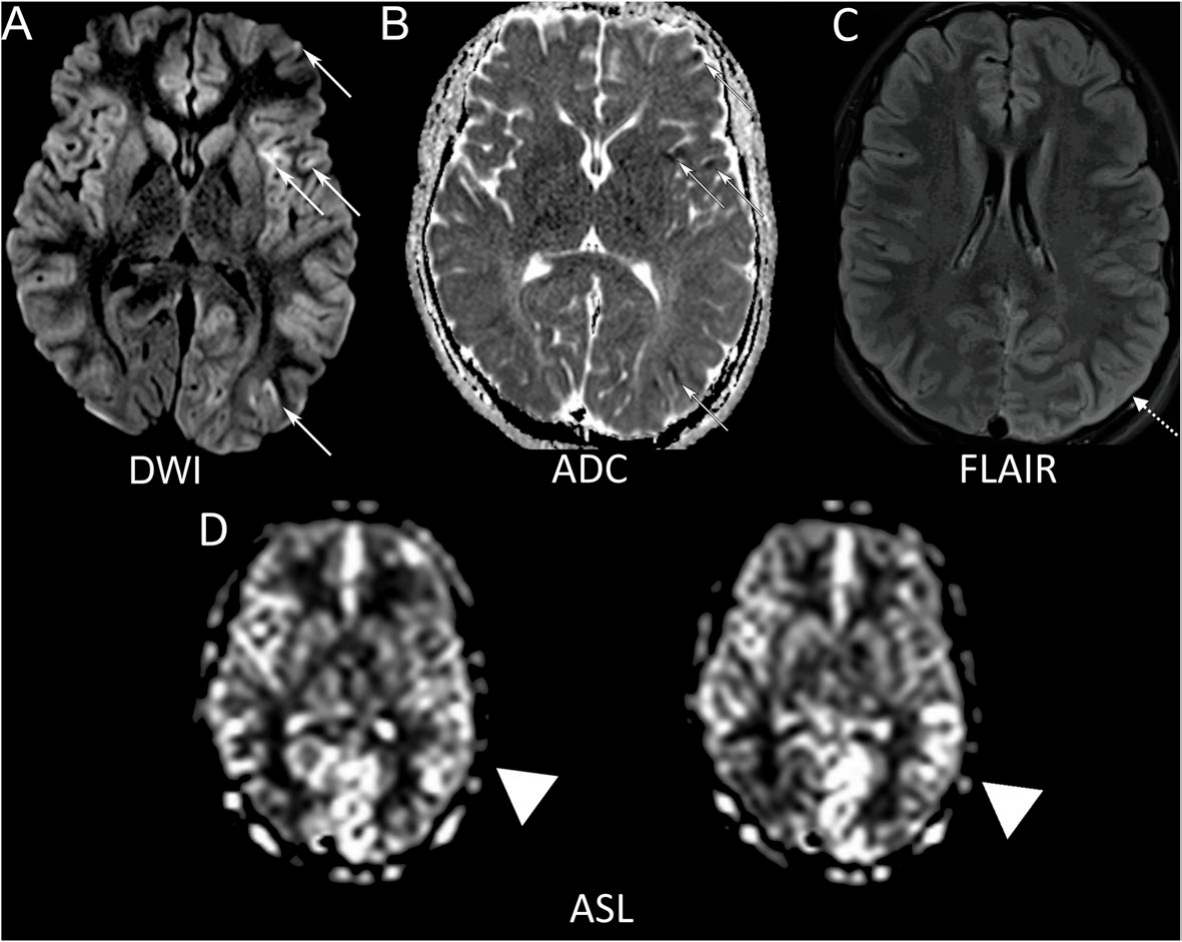
MRI brain demonstrates left hemispheric cortical restricted diffusion on trace diffusion weighted imaging (**A**—solid arrows) with correlate on apparent diffusion coefficient map (**B** – solid arrows). There is diffusely increased left cortical FLAIR signal (**C** – dot arrows) and areas of elevated perfusion on arterial spin labelled perfusion imaging (**D** –– arrowheads)

On the day of admission, the patient underwent unilateral ECT on the right side. Following ECT, they were noted to have right gaze deviation with right sided clonic activity and went into status epilepticus requiring rescue with high doses of lorazepam (26 mg total given in serial doses) and propofol 100 mg before arrival of emergency medical technicians (EMTs). They were intubated in the field, then brought to the emergency department (ED) and subsequently transferred to the pediatric intensive care unit (PICU) for ongoing care. On exam in the PICU, they were sedated and intubated with withdrawal of left sided extremities to noxious stimuli applied to the left side, but no movement of right sided extremities with noxious stimuli applied to the right side. They exhibited no seizures on EEG, but there was markedly asymmetric activity with significant slowing in the left hemisphere compared to the right. Screening infectious studies were unremarkable, which included completed blood count (CBC), comprehensive metabolic panel (CMP), blood culture, C-reactive peptide (CRP), and procalcitonin. They had a creatine kinase (CK) level drawn that was elevated to 1,121, which normalized over the course of 5 days. An initial MRI brain obtained one day after presentation to the ED was normal. Cerebrospinal fluid (CSF) analysis was unremarkable with normal cell counts, protein, and glucose levels. Testing for anti-NMDA receptor antibodies was negative. On day four, they developed spontaneous movement in the right leg, but the right arm remained completely plegic. In addition, their mental status improved, allowing for extubation. They had a repeat MRI on day four of admission that showed multifocal areas of cortical diffusion restriction in the left cerebral hemisphere and diffuse T2 prolongation in the cortex of the left hemisphere, more prominent posteriorly, as well as increased left hemispheric perfusion (Fig. 1). It is possible that the diffusion restriction is an artifact, but given the correlation with the T2 prolongation and clinical picture, it was thought to represent real signal changes. On day five of admission, strength on the right side improved, with near-normal function of the right leg, but persistent weakness in the right arm. They had antigravity movements of the right arm, proximally greater than distally. They demonstrated no cognitive abnormalities compared to baseline. The patient was discharged to an inpatient rehabilitation center for ongoing care and made a full neurologic recovery over the next two weeks. They continued to receive escalating doses of lorazepam to treat their catatonia during admission, which has since progressively improved. At an outpatient neurology visit three months following admission, they had normal strength to confrontation with mild difficulty with tandem gait as only objective finding on neurologic exam. A repeat MRI of their brain five months after presentation showed complete resolution of prior diffusion restriction, hyperemia, cerebral edema, and FLAIR abnormalities. They did not have any further episodes concerning for seizures. They had a whole-exome genetic sequencing analysis sent while admitted that resulted 4 months later and identified a heterozygote pathogenic *CACNA1a* variant (c.5126 T > C).

## Discussion and conclusion

*CACNA1a* encodes for the alpha-1A-subunit of the P/Q type calcium channel [4, 5]. These channels are expressed at central synapses and are particularly abundant in cerebellar granule and Purkinje cells [6–8]. They are also expressed at pre-synaptic terminals and modulate the synaptic release of neurotransmitters [9, 10].

Pathogenic variants in *CACNA1a* includes missense and nonsense point mutations, short and large deletions and tri-nucleotide repeat expansions. Single nucleotide pathogenic variants cause both gain-of-function and loss-of-function variants that have some correlation with phenotype. Clinically, pathogenic variants in *CACNA1*a span a spectrum of phenotypes that include familial hemiplegic migraine Type 1 (FHM1) and Episodic Ataxia Type 2 (EA2) with FHM1 being caused typically by gain-of-function variants and EA2 being caused by loss-of-function variants, although there can be overlap [11]. *CACNA1a* variants have also been associated with developmental delay, autism spectrum disorders, epilepsy and paroxysmal movement disorders (benign paroxysmal torticollis of infancy, paroxysmal tonic upgaze) [12–18].

Regarding the specific variant identified in this patient, the c.5126 T > C (*p*.I1709T) variant has been reported in patients with familial hemiplegic migraines with status epilepticus [19, 20]. Furthermore, there have also been prior reports of acute unilateral cerebral edema that can occur in patients with *CACNA1a* associated FHM1 in addition to other forms of familial hemiplegic migraines [21–23]. The pathologic mechanisms that lead to unilateral cerebral edema in familial hemiplegic migraine is unclear. However, one explanation is that the cortical spreading depression observed in FHM is associated with cerebral edema due to changes in ion gradients and decreased extracellular space due to cellular swelling with resultant hyperemia (increased perfusion) and diffusion restriction changes on MRI [24–27].

Most patients tolerate ECT well with the most common side effects including nausea, headaches, and brief periods of retrograde/anterograde amnesia [1, 28, 29]. Conventional bilateral ECT has been associated with a variety of transient neurological signs and symptoms, such as abnormal reflexes, aphasias, various agnosias, and Gerstmann syndrome. Transient hemiparesis or Todd paralysis was first reported with unilateral ECT in 1953, with other cortical dysfunction associated with the hemisphere stimulated, such as agnosia, sensation or visual changes, or language dysfunction. ECT in children and adolescents constitutes less than 1% of ECT in the United States [3, 30]. However, the most common side effects in this cohort—headache, memory problems, muscle soreness, and nausea/vomiting—are similar to the side effect profile in older patients [28, 29]. Another cohort analysis of ECT in adolescents reported prolonged seizures (defined as seizures longer than 3 min) in 4% (2 of 49 ECT treatments) of patients. There have been no published reports of prolonged focal neurologic deficits in adolescents treated with ECT.

In adult studies, unilateral ECT has previously shown hemisphere dependent deficits that typically resolve within 30 min (hemiplegia typically resolved by 15 min), but prolonged non-dominant hemisphere related deficits have been reported hours to days after treatment, but were largely visuo-spatial related deficits [31]. While deficits associated with unilateral ECT typically resolve within 30 min, some deficits have been reported hours to days after treatment. In one case report, a 58-year-old with depression received right-sided ECT and subsequently developed right facial droop, right upper and lower extremity weakness, and aphasia. Her motor symptoms resolved by 72 h, and her aphasia completely resolved by day five. An MRI obtained in this patient at day three following ECT was reportedly normal. The patient had previously received ECT without issue [32].

Regarding the imaging findings in this patient, although MRI has been used to evaluate structural changes related to long-term use of ECT and largely shows regional volumetric changes or differences in fractional anisotropy in different white matter tracts, there are few reports of MRI findings concerning for ischemia but none similar to the findings in this patient [33, 34]. There are rare reports of acute ischemic strokes following ECT, but our imaging pattern was not that of an arterial ischemic stroke [33, 34]. Overall, the MRI findings in the patient presented in this report are not consistent with reported imaging changes associated with ECT.

In conclusion, we present a case of a patient that developed prolonged right-sided hemiplegia following right sided ECT and subsequently found to have *CACNA1a* pathogenic variant. While ECT is generally a well-tolerated treatment based on published data, there is limited data on its use in children and in patients with specific genetic conditions. In the patient presented here, given their *CACNA1a* pathogenic variant, the episode of ECT potentially triggered a hemiplegic migraine and cortical spreading depression (CSD), which would be consistent with their MRI changes, cerebral edema and prolonged hemiplegia [35, 36]. It is not clear why ECT on the right hemisphere caused left sided symptoms, but during unilateral ECT, spread of seizures to contralateral side has been observed [31]. In this case, even though the ECT was on the right side, it seemed to trigger a seizure that also affected the left hemisphere based on the initial clinical semiology of this patient. In FHM associated cerebral edema, the imaging findings are typically unilateral which is not completely understood, but animal models have shown increased susceptibility to spreading depressions as a mechanism for cerebral edema. Of note, mild head trauma has been associated with some of these episodes which suggests that mild stressors may be triggers for hemiplegic migraine episodes [37]. Other potential diagnoses include a prolonged Todd’s paralysis related to their episode of status epilepticus, but their prolonged recovery and imaging findings are more consistent with an FHM episode given their underlying genetic disorder. Hemiplegia-hemiconvulsive-epilepsy is another entity with similar presentation, but variants in FHM related genes that include *CACNA1a* have been identified in some of these patients and typically these patients present during early childhood following febrile illness and go on to develop intractable epilepsy, which was not the case in the patient presented in this report [38].

This case highlights an unusual presentation of a patient with a pathogenic *CACNA1a* variant. In addition, the adverse events experienced by this patient suggest that care should be taken in patients undergoing ECT with *CACNA1a* related disorders. This case report also reinforces the role of genetic testing in patients with neurodevelopmental disorders as the results can help identify potential risk factors for side effects from specific treatments.

## Data Availability

Genetic variant identified in this patient has been uploaded to dbSNP database (accession number: rs121909326) and ClinVar database (accession number: VCV000008510.7) both of which can be accessed through NIH National Library of Medicine website.

## Abbreviations

ECT: Electroconvulsive therapy
MRI: Magnetic resonance imaging
CSD: Cortical spreading depression
FHM: Familial hemiplegic migraine
ED: Emergency department
EMT: Emergency medical technician
